# Implementing a medical student interpreter training program as a strategy to developing humanism

**DOI:** 10.1186/s12909-018-1254-7

**Published:** 2018-06-18

**Authors:** Alvaro F. Vargas Pelaez, Sarah I. Ramirez, Chavely Valdes Sanchez, Shady Piedra Abusharar, Jose C. Romeu, Connor Carmichael, Soraya Bascoy, Rose Baron, Ariana Pichardo-Lowden, Nathalia Albarracin, Claire C. Jones, Patricia Silveyra

**Affiliations:** 1 Department of Medicine, The New York University School of Medicine, New York, NY USA; 2 Department of Family and Community Medicine, Penn State College of Medicine, Hershey, PA USA; 3 Penn State College of Medicine, Hershey, PA USA; 4 Department of Medicine, Penn State College of Medicine, Hershey, PA USA; 5 Health Federation of Philadelphia, Philadelphia, PA USA; 6 Departments of Pediatrics, Biochemistry and Molecular Biology, and Humanities, Penn State College of Medicine, 500 University Drive, Mail Code H085, Hershey, PA 17033 USA

**Keywords:** Medical interpretation, Limited English proficiency patients, Medical students, Empathy, Humanism, Medical education

## Abstract

**Background:**

Humanistic care in medicine has shown to improve healthcare outcomes. Language barriers are a significant obstacle to humanistic care, and trained medical interpreters have demonstrated to effectively bridge the gap for the vulnerable limited English proficiency (LEP) patient population. One way in which medical schools can train more humanistic physicians and provide language access is through the implementation of programs to train bilingual medical students as medical interpreters. The purpose of this prospective study was to evaluate whether such training had an impact on bilingual medical student’s interpretation skills and humanistic traits.

**Methods:**

Between 2015 and 2017, whole-day (~ 8 h) workshops on medical interpretation were offered periodically to 80 bilingual medical students at the Penn State College of Medicine. Students completed a series of questionnaires before and after the training that assessed the program’s effectiveness and its overall impact on interpretation skills and humanistic traits. Students also had the opportunity to become certified medical interpreters.

**Results:**

The 80 student participants were first- to third- year medical students representing 21 languages. Following training, most students felt more confident interpreting (98%) and more empathetic towards LEP patients (87.5%). Students’ scores in the multiple-choice questions about medical interpretation/role of the interpreter were also significantly improved (Chi-Square test, *p* < 0.05). All students who decided to take the exam were able to successfully become certified interpreters. Ninety-two percent of participants reported they would recommend the program and would be willing to serve as a future “coaches” for interpreter training workshops delivered to peer students.

**Conclusions:**

Our program was successful in increasing self-reported measures of empathy and humanism in medical students. Our data suggests that implementation of medical interpreter training programs can be a successful strategy to develop of humanism in medical students, and aid in the development of sustainable language access for LEP patients.

**Electronic supplementary material:**

The online version of this article (10.1186/s12909-018-1254-7) contains supplementary material, which is available to authorized users.

## Background

Humanism in medicine, as per the Arnold P. Gold Foundation, is characterized by a respectful and compassionate relationship between the healthcare team and patients [1]. It reflects attitudes and behaviors of the team members that are sensitive to the values and the cultural and ethnic backgrounds of others [2, 3]. Research has shown that when physicians provide humanistic care, patients are more likely to adhere to medical advice and treatment options, resulting in improved health outcomes [4]. Similarly, cultural competency, defined as “a set of congruent behaviors, attitudes, and policies that come together in a system, agency, or among professionals that enables effective work in cross-cultural situations”, has been identified as a major determinant of patient satisfaction, adherence, and outcomes [5]. Accordingly, it is imperative that medical education curriculum provide students with activities that will aid in preservation of empathy and humanism, and increase cultural competency, while preparing future physicians for understanding social, economic, and cultural factors that may affect the quality of the care they will provide.

An effective communication skillset is a critical component for creating close, empathetic relationship with patients and enabling effective work in cross-cultural situations [6]. However, effective communication is significantly more challenging when patients are not proficient in English. According to the US Census Bureau, it is estimated that more than 25 million of Americans speak English “less than very well” [7, 8]. These individuals are defined as having limited English proficiency (LEP) and have been shown to have lower health literacy, access to care, and satisfaction with the health care system [9, 10].

Studies suggest that racial and ethnic minorities are disproportionately affected by communication barriers associated with LEP and low health literacy [11–13]. These patients are at greater risk of being misunderstood by physicians, thus increasing their diagnosis time and re-admission rates, and reducing patient safety, compliance, and satisfaction [14–20]. Ensuring LEP patients can effectively provide physicians with a clear statement of their medical condition, and understand the provider’s assessment and treatment options, is essential to the provision of quality patient care and compliance with the title VI of the Civil Rights Act of 1964 [21]. To mitigate these issues the Joint Commission and the United States Department of Health and Human Services Office of Minority Health provide national standards for Culturally and Linguistically Appropriate Services (CLAS) in Health Care to facilitate appropriate services for LEP patients at no cost and in their primary language. [22, 23]

Of note, given the value of the spoken word in effective communication, interpretation services, which by definition convert oral messages only, are the most needed (in contrast with translation services, which converts written messages). In general, interpretation services at medical centers are available via Dual-Handset phones or in-person interpretation. In-person interpretation usually requires prior scheduling and due to the low availability of trained medical interpreters and time constrains, providers tend to choose this method less frequently [24].

The use of professional medical interpreters has been demonstrated to help bridge the gap for LEP patients [22, 25–27]. The benefits of professional medical interpreting include improved comprehension, fewer communication errors, better care and compliance, increased patient satisfaction, lower risk of adverse events, lower re-admission rates, and lower malpractice risk [28–30]. Studies suggest that in-person interpretation is more efficient as it includes non-verbal cues that facilitate transmission of information [31, 32]. Moreover, interpreters can serve as “cultural brokers”, allowing patients to focus on their healthcare concerns [33]. Healthcare organizations have started to increase the number of trained in-person medical interpreters available to patients, either by hiring more medical interpreters, contracting with outsourcing companies, and/or by providing training to their bilingual staff [25]. As the nation’s landscape trends towards the “minority becoming the majority” and the number of bilingual and diverse providers fails to grow accordingly, training medical students on the role of medical interpreters will help address future needs of the healthcare system [16, 34, 35]. In addition, this training would also provide students with components of cultural competency and humanism in medicine that are not typically included in the medical school curriculum [36, 37].

In recent years, the number of bilingual medical students has significantly increased, due to concomitant increases in access to college education for second-generation immigrants and student engagement in language programs and medical linguistics courses [38]. Despite numerous successful programs which have effectively trained medical and undergraduate students to work as or with medical interpreters, to our knowledge there are no studies which explore the effect of these trainings as education tools to enhance medical humanism [34, 36, 37, 39]. We aimed to address this gap by implementing a medical interpretation skills training program tailored for medical students that included the core components of the interpretation process from traditional programs, combined with practice hours in simulated scenarios. In addition to evaluating the competency of students in terms of their interpretation skills and knowledge, we evaluated the effectiveness of the training in promoting humanism and empathy in the clinical curricular years. Our working hypothesis was that training students in medical interpretation skills enhances their communication abilities and empathy, resulting in improved humanism.

## Methods

### Study design and population

This was a prospective study in first- to third- year medical students. Between the spring of 2015 and the summer of 2017, whole-day (~ 8 h) workshops on medical interpretation were offered periodically to 80 medical students at the Penn State College of Medicine. Students were recruited via e-mail, students’ class Facebook pages, and directly by student leaders of organization chapters which tend to have bilingual presence such as the Latino Medical Student Association.

### Surveys and measures

Anonymous pre- and post- surveys were administered with questions about knowledge of interpretation and aspects of the interpreter’s role, cultural competencies, and information about their language skills and cultural background (Additional file 1). Only non-identifiable data were collected. All data were recorded on RedCap. The study was exempted from review by the Penn State College of Medicine Institutional Review Board.

### Workshop details

The program was fully consistent with the standards and goals set by The National Board of Certification for Medical Interpreters (Table 1) and taught in conjunction with a representative of the Health Federation of Philadelphia, a non-profit organization which provides interpreter training in the State of Pennsylvania [40]. In each module, learning was experiential, and participants had numerous opportunities to self-assess and practice skills through practice-based role-plays, case studies and small group activities.

**Table 1:** Core content of the interpreter skills training course

Interpreter Roles and Responsibilities/Code of Ethics	
To distinguish between interpretation & translation	
To define & demonstrate consecutive & simultaneous interpretation	
To describe the interpreter’s role as “the transmitter of communication”	
To identify interpreter role boundaries	
To name the basic elements of the Interpreter Code of Ethics: accuracy, completeness, impartiality, confidentiality, discretion, professionalism	
Cultural Competencies and Organizational Protocols	
To define culture, cultural competency & culture brokering	
To explore professional culture and how it impacts service delivery and interpreting	
To explore the impact of culture on attitudes and beliefs about the practice area	
To identify & explain Culturally and Linguistically Appropriate Services Standards	
Basic Vocabulary & Interpretation Techniques and Issues	
To demonstrate proper “setting the stage” for the interpreted encounter (introductions and ground rules)	
To use bilingual practice vocabulary commensurate with protocols and anticipated interpretation assignments To apply interpretation strategies: paraphrasing, clarifying techniques, shadowing & interjection	
To practice ethical interpretation in routine and difficult situations, with special emphasis on the “dual role”, including the impact of trauma and vicarious trauma	

A variety of presentation formats that respect the learning preferences and multiple intelligences that diverse students bring to the table were considered. In addition, respectful and constructive feedback was provided throughout the training. At the end of the workshop, students were assigned to working groups based on language, and provided with time to acquire interpretation practice hours by role-playing with peers who spoke the same language, followed by a debriefing session. On average, training and practice hours totaled 10–15 h per student. Students were provided with the opportunity of taking a test to become certified medical interpreters.

### Assessments

The examination consisted of a behavioral simulation model using standardized situational role-plays tailored to the practice setting, in which interpretation skills were assessed orally. In these role-plays, trainees served as interpreters for a simulated patient and an English-speaking service provider. An example of a role-play can be seen in this video (https://www.youtube.com/watch?v=7FH9vS06rJ8). The lead trainer and training assistants observed and/or participated as actors in these role plays and scored the trainees on established criteria of performance including accuracy of interpretation and application of specific techniques.

The exam also included an important component about diverse cultural scenarios and management of difficult situations. All trainees were given feedback and specific recommendations for professional development. Great emphasis was placed in our training on CLAS standards, cultural competency, and practice of specialized skills and techniques for cross-cultural interviewing (Table 2).

**Table 2:** Training components presented on the medical interpreter exam

Ethics and Personal Responsibility	
Demonstrate respect, integrity, and professionalism for individuals and their communities.	
Global Awareness and Valuing Diversity	
Discuss how different cultural views may affect patient’s expectations of the interpreter.	
Explain Western medical culture and providers.	
Discuss how different cultural views may affect a patient’s expectations of the interpreter.	
Communication	
Describe confidentiality of the Institution’s Standards for Healthcare Interpreters.	
Identify health information protected by federal and state medical privacy and confidentiality laws (e.g. HIPAA).	
Critical Thinking	
Be able to describe proper cultural context in interpretation of medical terminology.	
Self-Awareness and Interpersonal Skills	
Analyze cultural background (your own/others), level of acculturation, personal beliefs, and values.	
Identify assumptions of cultural beliefs, values, and behaviors.	

Following the exam, students were administered a post-training assessment survey asking about their empathy towards LEP patients and English-only providers, and their willingness to advocate for LEP patients and for system change. Participants were also asked to provide written feedback about the program and their self-rated confidence about their interpretation abilities and perceived understanding of their role as interpreters (Additional file 2). Finally, participants were provided a post-exam anonymous survey asking to rate their perceived change in empathy towards LEP patients, English-only speaking providers, and their willingness to advocate for patients/physicians and system change as a result of the program (Additional file 3).

### Data analysis

Survey data were entered on RedCap, removing any potential identifiers. We used descriptive statistics to assess the study population in terms of language characteristics and distribution amongst participants, and previous experience interpreting prior to the training, and post-training self-assessments of empathy and inclination to advocate for change. To assess the effects of the training on the student’s ability to correctly answer questions about interpretation skills, we performed chi-square analyses on contingency tables using the GraphPad Prism version 7.0c for Mac OS X, GraphPad Software, La Jolla California USA, www.graphpad.com

## Results

### Student population, languages represented, and prior experience

The 80 student participants were fully bilingual first- to third- year medical students at our institution. Twenty-one languages were represented (Table 3). Several students were fluent in more than one language/dialect in addition to English. Only 31% of the students identified English as their first language, and 82% indicated they have worked, studied, or lived in a country other than the United States (Table 4). Most students learned the languages for which they sought interpreter certification at home/from their parents (49%) and/or at school (27%) (Table 5).

**Table 3:** Languages spoken by students

Represented Languages	% of students
Arabic	6
Burmese	1
Cantonese	1
Chinese	2
Creole	3
French	7
German	1
Gujaratu	2
Hindi	5
Korean	8
Malay	1
Mandarin	12
Marathi	1
Persian/Farsi	2
Polish	1
Portuguese	4
Spanish	43
Taiwanese	1
Thai	1
Urdu	1
Vietnamese	5

**Table 4:** Students’ background and exposure to medical interpretation

English is my first language	Yes	No
	31%	67%
I have lived/worked/studied in a country other than the US	82%	18%
I have had formal training interpreting prior to this training	5%	95%
I have interpreted as a volunteer for community/healthcare organizations	55%	45%
I have interpreted for family/friends	79%	21%

**Table 5:** Environment in which students acquired language proficiency

I have learned to speak my interpreting language at:	% of students
School	27%
home/ from my parents	49%
Other	24%

Even though 95% of the students had never received formal interpreting training, 55% of them had interpreted as volunteers for a community/healthcare organization, and 79% had interpreted for family/friends in the healthcare setting (Table 4).

### Effect of training on student’s confidence and empathy towards LEP

**Fig. 1:**
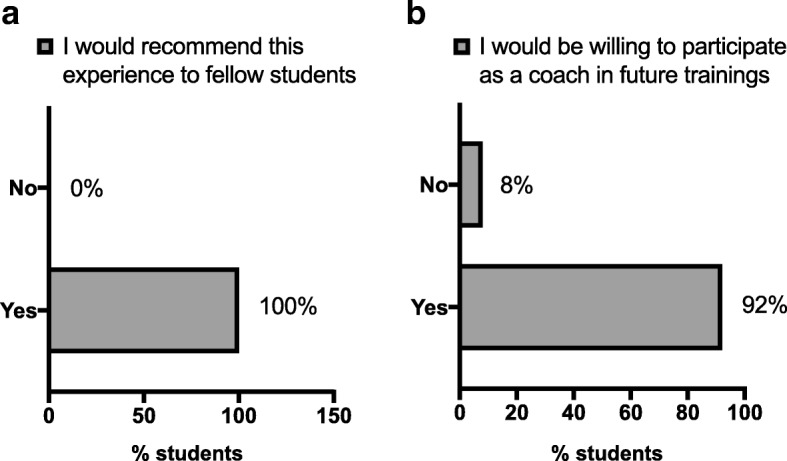
Data obtained from students who responded to anonymous post-surveys (*n* = 80). Histograms represent the percentage of students who reported that they would recommend this training and experience to other fellow students (**a**), and students who reported that they would be willing to serve as a “coach” for future training sessions (**b**)

As a result of the training, almost all students (98%) indicated that they felt more confident with regards to interpreting (Table 6). The great majority of students (87.5%) also felt more empathetic towards LEP patients and towards English-only providers (71%) (Table 6), indicating greater empathy for individuals affected by the communication barrier (patients and providers). Additionally, students reported feeling more inclined to advocate for patients (77%) and to work for system change (84%) (Table 6). Finally, the program was very well-received by students, with all participants reporting they would recommend this training and experience to other fellow students and 92% reported that they would be willing to serve as a “coach” for future training sessions (Fig. 1a-b).

**Table 6:** Students’ responses after completing the training program

Confidence about interpreting	More	Less	Same
	98%	0%	2%
Empathy for LEP patients	87.5%	0%	12.5%
Empathy for English-only providers	71%	0%	29%
Inclined to advocate for patients	77%	4%	19%
Inclined to work for system change	84%	2%	14%

### Effect of training on student’s understanding of the interpretation process and empathy

**Fig. 2:**
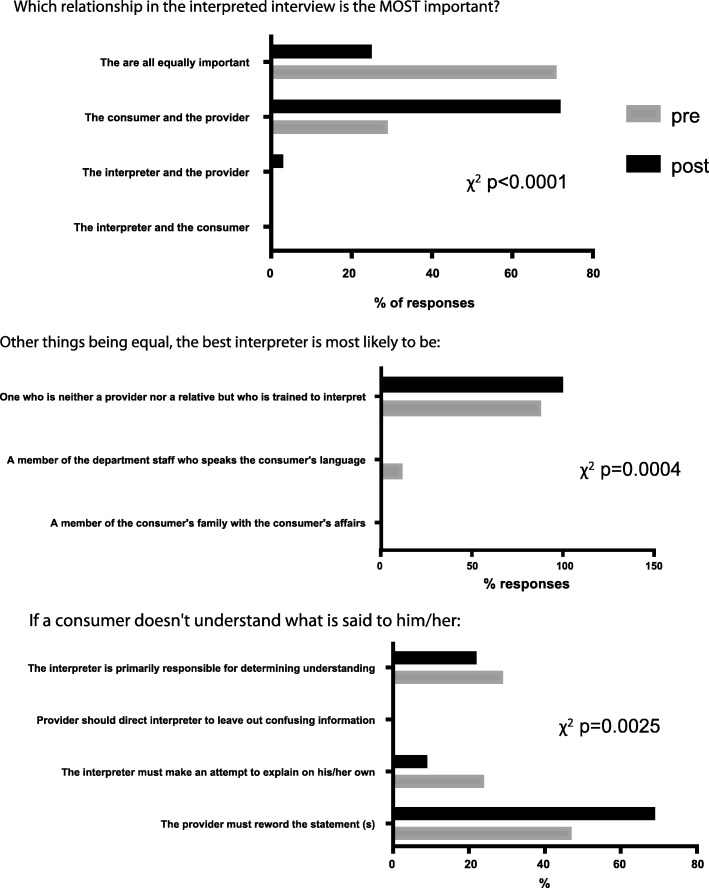
Difference in student’s answer selections in multiple choice questions about the medical interpretation process and the interpreter’s role in pre- and post- training surveys (n = 80, χ^2^ *p*-values are indicated for each question)

**Fig. 3:**
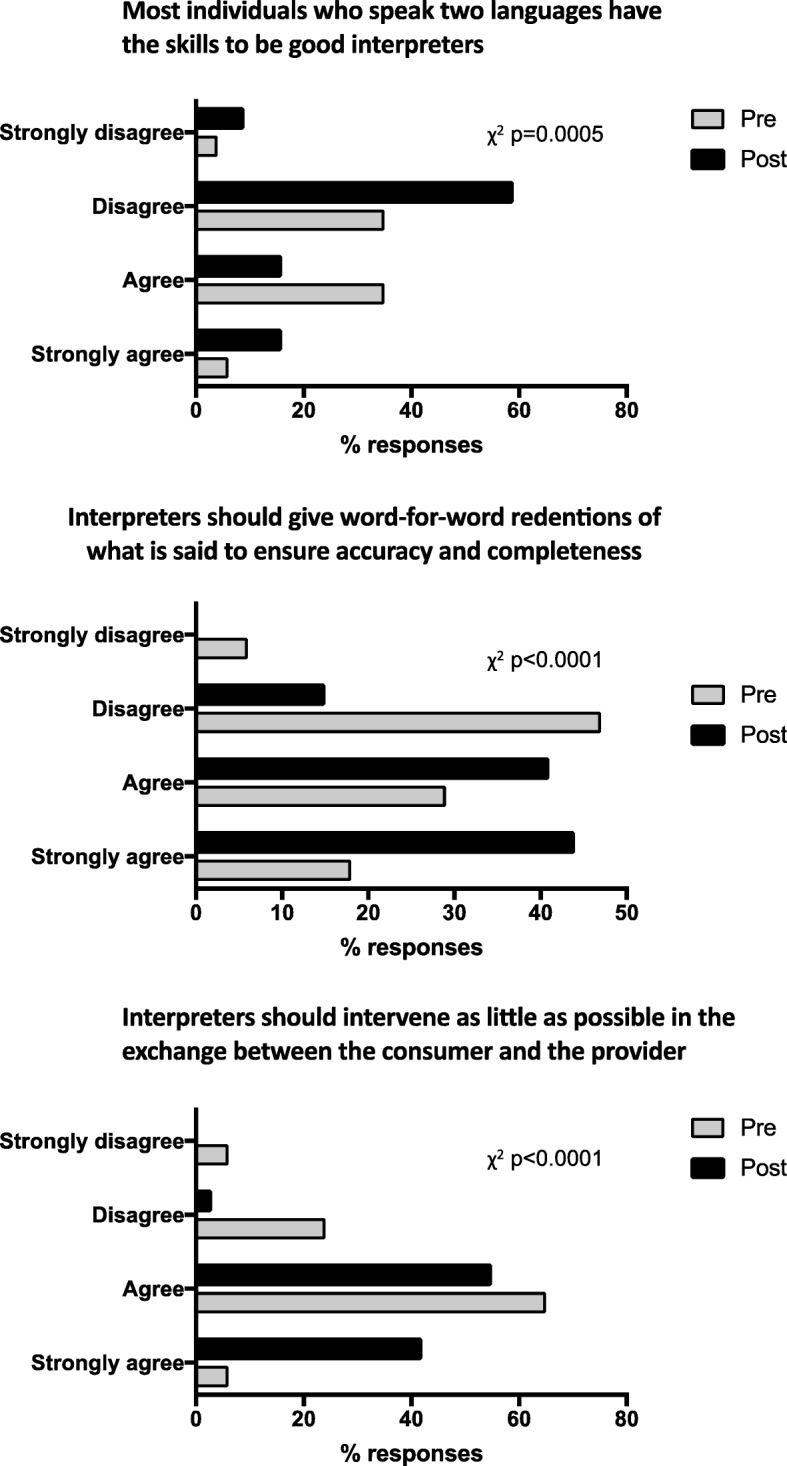
Difference in student’s answer selections in multiple choice questions about the interpreter’s role in pre- and post- training surveys (*n* = 80, χ^2^ p-values are indicated for each question)

**Fig. 4:**
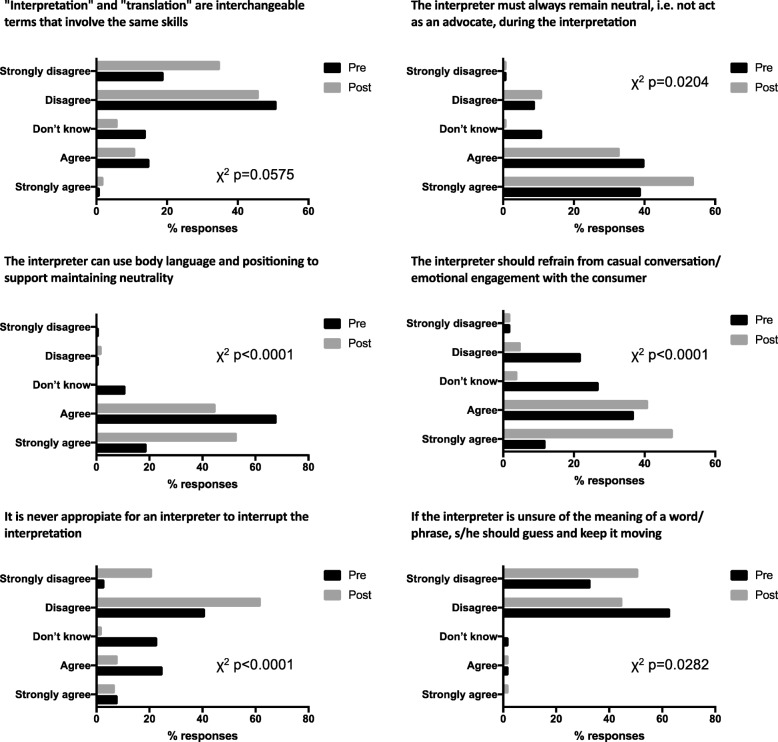
Difference in student’s answer selections in multiple choice questions with regards to the medical interview process in pre- and post- training surveys (*n* = 80, χ^2^ p-values are indicated for each question)

**Fig. 5:**
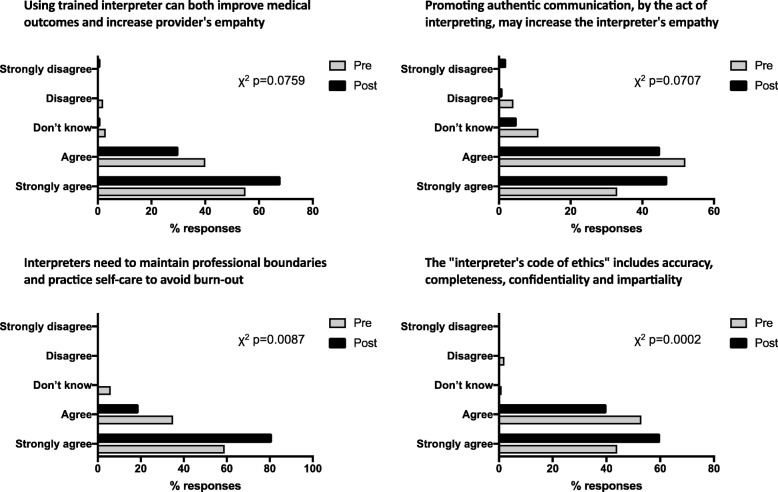
Difference in student’s answer selections in multiple choice questions with regards to various aspects of empathy and professionalism in pre- and post- training surveys (*n* = 80, χ^2^ p-values are indicated for each question)

As a group, the overall students’ scores in multiple choice questions about the medical interpretation process and the interpreter’s role were significantly improved as a result of the training (χ^2^ *p*-values < 0.05) (Fig. 2). In the Likert-type scale questions about the interpreter’s role and professionalism, students showed significant improvement towards the correct scale choices about the interpreter’s role (Fig. 3). Similar results were observed in questions about the medical interview process (Fig. 4), as well as various aspects of empathy and professionalism (Fig. 5).

## Discussion

Although medical school curricula are designed to address the broad and changing healthcare needs of society, studies have suggested that medical education can lead to deterioration of empathy among students, especially in the clinical years, when the curriculum increases in intensity and shifts towards patient-care activities [41]. Numerous educators have reported that this decline could be mitigated or reversed by implementing activities and programs designed to sustain humanism in medicine, and to help students preserve sensitivity to the humanity of their patients [42–44]. Similarly, language and cultural barriers create significant health disparities and are important obstacles in the creation of empathetic therapeutic relationships, particularly among LEP patients [17–19]. Research has shown that professionally-trained medical interpreters can help mitigate these obstacles, and for this reason there is a great need to develop programs to increase the number of trained medical interpreters and also educate future physicians to provide culturally competent medical care and understand issues pertaining to communication with patients who have LEP [28–30]. The aim of this study was to address these issues by implementing a medical interpretation training program with bilingual medical students with the goal of creating a sustainable language access program and increasing students’ communication skills and empathy, potentially resulting in more humanistic medical professionals.

At the end of the program, students demonstrated to be proficient medical interpreters. They were confident in their abilities and knowledgeable about the basic concepts necessary for providing effective medical interpretation, such as understanding of the role, proper position, and duties of the interpreter. Our students also reported feeling more empathetic towards LEP patients, English-only providers, and feeling more inclined to advocate for LEP patients and system change.

Interpreting is a value-added skill that enhances bilingual healthcare communication, and requires extreme professionalism. Therefore, it is not surprising healthcare organizations can be hesitant to use students as interpreters. However, medical students are a very select group with high education level, healthcare literacy, and motivation, and are in an ideal position to provide this service. To our knowledge, this is the first program which provides students the opportunity of becoming certified interpreters. While there are many competing certification organizations for medical interpreters and the length/content of training varies, students reported that having an official certification was an important factor to allow them to serve within our institution and the local community, thereby increasing the interpretation workforce in compliance with hospital policies [45].

There were several challenges, limitations, and lessons learned during this project. First, unsurprisingly, medical students are very busy and it was difficult to schedule post-training practice hours to fully comply with the original 40-h training design, although most students received between 15 and 20 h of training, which was shown to be effective according to our results. We modified the program to reduce lecture and increase practicing time, allowing students to take the certification test the day of the training. Despite this significantly decreased number of supervised training hours, our data and previous work suggest that shorter durations of training can potentially be as effective in this particular population [37]. Secondly, we only used self-rated levels of empathy. In this regard, future studies could also incorporate patients and/or providers’ perceptions of trained students compared to untrained students. Thirdly, this program only captured bilingual medical students. Other interventions have been identified for non-bilingual students, as all students should be targeted [36]. Finally, we did not include a control group, nor did we compare our trained interpreters with other professional/trained interpreters. Our group is currently piloting several projects in which medical student interpreters help the hospital floors, diabetes education groups, and walk-in clinics, where both staff and patients have the opportunity to compare among different types of interpreters/interpretation modalities and their effectiveness in addressing some of these issues.

In summary, the United States’ population is rapidly becoming more linguistically and culturally diverse; and despite great efforts, the gap between the growing LEP population and multilingual physicians continues to widen [35]. In addition to imparting medical knowledge and skills, medical schools have a professional and ethical duty to prepare the next generation of healthcare professionals to be competent at caring and recognizing humanity in their patients beyond any linguistic or cultural barriers. Similarly, healthcare organizations are legally obligated to provide advanced resources such as free-of-charge language services to vulnerable populations such as LEP patients. Our work presented here suggests that medical schools (and potentially other health professional schools as well) can possibly help address these two important issues through the implementation of medical interpretation training programs.

## Conclusions

The results from our study suggest that implementation of medical interpreter training programs can be a successful strategy to develop of humanism in medical students, and can aid in the development of more comprehensive and culturally inclusive language access for LEP patients. Additional investigation of coordinated programs is needed to evaluate the sustainability of these types of interpretation programs, not only in medical students, but also in other healthcare careers. We strongly believe that by incorporating high-quality medical interpretation training in the medical student curriculum, schools can form culturally versed and empathetic physicians and provide better care for diverse populations.

## Additional files

Additional file 1: Anonymous pre-workshop surveys (“Part 1”, “Part 2 pre”, and “Part 3”), and post-workshop surveys (“Part 1” and “Part 3”) administered to participants to obtain information about the interpreation process knowledge, cultural competencies, language skills and cultural background . (PDF 55 kb)

Additional file 2: Anonymous survey (“Part 2 post”) administered to workshop participants to obtain post-workshop feedback about the program and their self-rated confidence about their interpretation abilities. (PDF 38 kb)

Additional file 3: Anonymous post-workshop survey (“Exam evaluation”) administered to evaluate participant’s perceived change in empathy towards patients and providers, and advocacy aspirations. (PDF 40 kb)

## Abbreviations

CLAS: Culturally and Linguistically Appropriate Services
LEP: Limited English proficiency
