# Supplementary material for: Implementing a medical student interpreter training program as a strategy to developing humanism
Source: BMC Med Educ. 2018 Jun 18;18:141. doi: 10.1186/s12909-018-1254-7 (PMC6006684; doi:10.1186/s12909-018-1254-7)
Supplement: Supplementary file 1 — Anonymous pre-workshop surveys (“Part 1”, “Part 2 pre”, and “Part 3”), and post-workshop surveys (“Part 1” and “Part 3”) administered to participants to obtain information about the interpreation process knowledge, cultural competencies, language skills and cultural background . (PDF 55 kb) [file 12909_2018_1254_MOESM1_ESM.pdf]

Part 1

Participant ID ______________________

The interpreter's role is to facilitate communication between the parties to a conversation
- ◯ Strongly Agree
- ◯ Agree
- ◯ Don't Know
- ◯ Disagree
- ◯ Strongly Disagree

The interpreter is responsible for determining whether a consumer understands what the provider says
- ◯ Strongly Agree
- ◯ Agree
- ◯ Don't Know
- ◯ Disagree
- ◯ Strongly Disagree

The interpreter must consider grammar, syntax and register when interpreting
- ◯ Strongly Agree
- ◯ Agree
- ◯ Don't Know
- ◯ Disagree
- ◯ Strongly Disagree

If a provider asks the interpreter to simply summarize what is said, s/he should comply
- ◯ Strongly Agree
- ◯ Agree
- ◯ Don't Know
- ◯ Disagree
- ◯ Strongly Disagree

If a consumer or provider asks the interpreter to leave something out, s/he should comply
- ◯ Strongly Agree
- ◯ Agree
- ◯ Don't Know
- ◯ Disagree
- ◯ Strongly Disagree

The preferred method for interpreting for healthcare and human services is "simultaneous interpretation"
- ◯ Strongly Agree
- ◯ Agree
- ◯ Don't Know
- ◯ Disagree
- ◯ Strongly Disagree

"Interpretation" and "translation" are interchangeable terms that involve the same skills
- ◯ Strongly Agree
- ◯ Agree
- ◯ Don't Know
- ◯ Disagree
- ◯ Strongly Disagree

The interpreter must always remain neutral, i.e. not act as an advocate, during the interpretation
- ◯ Strongly Agree
- ◯ Agree
- ◯ Don't Know
- ◯ Disagree
- ◯ Strongly Disagree

The interpreter can use body language and positioning to support maintaining neutrality
- ◯ Strongly Agree
- ◯ Agree
- ◯ Don't Know
- ◯ Disagree
- ◯ Strongly Disagree

The interpreter should refrain from casual conversation/emotional engagement with the consumer

◯ Strongly Agree
◯ Agree
◯ Don't Know
◯ Disagree
◯ Strongly Disagree

It is never appropriate for an interpreter to interrupt the interpretation

◯ Strongly Agree
◯ Agree
◯ Don't Know
◯ Disagree
◯ Strongly Disagree

If the interpreter is unsure of the meaning of a word/phrase, s/he should guess and keep it moving

◯ Strongly Agree
◯ Agree
◯ Don't Know
◯ Disagree
◯ Strongly Disagree

The interpreter may use electronic or hard copy dictionaries as much as needed during the interpretation

◯ Strongly Agree
◯ Agree
◯ Don't Know
◯ Disagree
◯ Strongly Disagree

The interpreter should request information about the nature of the consumer's appointment beforehand

◯ Strongly Agree
◯ Agree
◯ Don't Know
◯ Disagree
◯ Strongly Disagree

It is never appropriate for the interpreter to inquire about cultural beliefs during the interpretation

◯ Strongly Agree
◯ Agree
◯ Don't Know
◯ Disagree
◯ Strongly Disagree

The most important relationship (affect) in an interpretation is between consumer and provider

◯ Strongly Agree
◯ Agree
◯ Don't Know
◯ Disagree
◯ Strongly Disagree

Using a trained interpreter can both improve medical outcomes and increase provider's empathy

◯ Strongly Agree
◯ Agree
◯ Don't Know
◯ Disagree
◯ Strongly Disagree

Promoting authentic communication, by the act of interpreting, may increase the interpreter's empathy

◯ Strongly Agree
◯ Agree
◯ Don't Know
◯ Disagree
◯ Strongly Disagree

Interpreters need to maintain professional boundaries and practice self-care to avoid burn-out

◯ Strongly Agree
◯ Agree
◯ Don't Know
◯ Disagree
◯ Strongly Disagree

The "interpreter's code of ethics" includes accuracy, completeness, confidentiality and impartiality

◯ Strongly Agree
◯ Agree
◯ Don't Know
◯ Disagree
◯ Strongly Disagree

Confidential

Part 2 pre

As a participant in this study, please answer the questions below. Thank you!

I have had formal training interpreting prior to this training	◯ Yes<br>◯ No
I have interpreted for family/friends	◯ Yes<br>◯ No
If yes, how frequently?	__________________________
I have interpreted as a volunteer for community/healthcare organizations	◯ Yes<br>◯ No
If yes, how frequently?	__________________________
English is my first language	◯ Yes<br>◯ No
The language(s) other than English in which I am fluent is (are)	__________________________<br>((list all that apply))
I learned to speak it (them)	☐ school<br>☐ at home<br>☐ from my parents<br>☐ other
please specify	__________________________
at what age?	__________________________
I read and write the following language(s) other than English at a 9th grade or higher level	__________________________<br>((list all that apply))
I have lived/worked/studied in a country other than the US	◯ Yes<br>◯ No
If yes, what country(ies)	__________________________

Part 3

State at least 4 things you can do to "set the stage" for interpreting to begin

____

What 2 actions must you take when the consumer asks you to not tell the provider something

____

Name 3 techniques you can use when you have a general idea of the meaning of something that has been said to you, but you are not completely sure

____

What do you do when you do not like the consumer or the provider?

____

List 4 ways you can manage the flow of communication during the interpretation

____

What 2 steps should you take when the consumer and provider are misscommunicating or don't understand each other

____

Describe the role of an interpreter. What does he/she do?

____

Give 3 situations in which you should interrupt the interpretation and provide your own suggestions. How often should you interrupt?

____

Name at least 4 of the skills that a person needs to be able to work as an interpreter

____

State 4 reasons why a code of ethics for interpreters is necessary

____

Which relationship in the interpreted interview is the MOST important

- ◯ the interpreter and the consumer
- ◯ the interpreter and the provider
- ◯ the consumer and the provider
- ◯ they are all equally important

During an interpretation session, you should stand (or sit)

- ◯ next to the consumer
- ◯ next to the provider
- ◯ midway between them
- ◯ out of sight of them

Which type of interpretation is recommended for healthcare and human services

- ◯ consecutive
- ◯ simultaneous
- ◯ escort

Other things being equal, the best interpreter is most likely to be

- ◯ a member of the consumer's family who is familiar with the consumer's affairs
- ◯ a member of the department staff who speaks the consumer's language
- ◯ one who is neither a provider nor a relative but who is trained to interpret

During the medical interview, an interpreter should be expected to

- ◯ advocate for the consumer
- ◯ advocate for the provider
- ◯ advocate for both
- ◯ advocate for neither

A well-trained and competent interpreter

- ◯ should be ready to go when the interview starts without needing any background
- ◯ should maintain a neutral background role as much as possible
- ◯ should never interrupt the interview
- ◯ all above are correct

If a consumer doesn't understand what is said to him/her

- ◯ the provider must reword the statement(s)
- ◯ the interpreter must make an attempt to explain on his/her own
- ◯ the provider should direct the interpreter to leave out confusing information
- ◯ the interpreter is primarily responsible for determining understanding

Which one of these statements is true?

- ◯ interpretation is repeating what is said word-for-word, and translating is repeating a general idea
- ◯ interpretation involves deciding which language issues, cultural beliefs and consumer needs are important for the consumer and explaining them to the provider
- ◯ translation is changing written communication from one language to another language
- ◯ all these statements are true

Knowledge of technical vocabulary in both working languages us the single most important element for interpreters

- ◯ Strongly agree
- ◯ Agree
- ◯ Disagree
- ◯ Strongly disagree

Interpreters are instruments of communication, but not participants in the task to be done by the worker (doctor, nurse, social worker, etc.)

- ◯ Strongly agree
- ◯ Agree
- ◯ Disagree
- ◯ Strongly disagree

Interpreters should give word-for-word renditions of what is said to ensure accuracy and completeness

- ◯ Strongly agree
- ◯ Agree
- ◯ Disagree
- ◯ Strongly disagree

Interpreters should intervene as little as possible in the exchange between the consumer and the provider

- ◯ Strongly agree
- ◯ Agree
- ◯ Disagree
- ◯ Strongly disagree

Most individuals who speak two languages have the skills to be good interpreters

- ◯ Strongly agree
- ◯ Agree
- ◯ Disagree
- ◯ Strongly disagree

Developing endurance enhances an interpreter's performance

- ◯ Strongly agree
- ◯ Agree
- ◯ Disagree
- ◯ Strongly disagree

Use of hand gestures can improve the flow of interpretation

- ◯ Strongly agree
- ◯ Agree
- ◯ Disagree
- ◯ Strongly disagree
