# Supplementary material for: Implementing a medical student interpreter training program as a strategy to developing humanism
Source: BMC Med Educ. 2018 Jun 18;18:141. doi: 10.1186/s12909-018-1254-7 (PMC6006684; doi:10.1186/s12909-018-1254-7)
Supplement: Supplementary file 2 — Anonymous survey (“Part 2 post”) administered to workshop participants to obtain post-workshop feedback about the program and their self-rated confidence about their interpretation abilities. (PDF 38 kb) [file 12909_2018_1254_MOESM2_ESM.pdf]

Part 2 post

The presentation held my interest

◯ Strongly Agree
◯ Agree
◯ Don't Know
◯ Disagree
◯ Strongly Disagree

The content, style and pace of this presentation was appropriate

◯ Strongly Agree
◯ Agree
◯ Don't Know
◯ Disagree
◯ Strongly Disagree

There was ample time to practice new skills

◯ Strongly Agree
◯ Agree
◯ Don't Know
◯ Disagree
◯ Strongly Disagree

Example handouts, and case studies were relevant and easy to understand

◯ Strongly Agree
◯ Agree
◯ Don't Know
◯ Disagree
◯ Strongly Disagree

This training increased my understanding of the interpreter's role on the healthcare team

◯ Strongly Agree
◯ Agree
◯ Don't Know
◯ Disagree
◯ Strongly Disagree

My ability to facilitate communication between providers and consumers is greater as a result of this training

◯ Strongly Agree
◯ Agree
◯ Don't Know
◯ Disagree
◯ Strongly Disagree

My confidence in facilitating communication between providers and consumers is greater as a results of this training

◯ Strongly Agree
◯ Agree
◯ Don't Know
◯ Disagree
◯ Strongly Disagree

I still have questions about (please list)

____

I would like more information about (please list)

____

Additional comments

____
