# Supplementary material for: Implementing a medical student interpreter training program as a strategy to developing humanism
Source: BMC Med Educ. 2018 Jun 18;18:141. doi: 10.1186/s12909-018-1254-7 (PMC6006684; doi:10.1186/s12909-018-1254-7)
Supplement: Supplementary file 3 — Anonymous post-workshop survey (“Exam evaluation”) administered to evaluate participant’s perceived change in empathy towards patients and providers, and advocacy aspirations. (PDF 40 kb) [file 12909_2018_1254_MOESM3_ESM.pdf]

Exam evaluation

My "other" language is ________________________________

This was my first experience in interpreting
◯ Yes
◯ No

I had interpreter training prior to this class/practicum
◯ Yes
◯ No

I had opportunities to practice after the training with fellow students
◯ Yes
◯ No

I had opportunities to practice after the training with my professor
◯ Yes
◯ No

other (whom?)
◯ Yes
◯ No

____

I had opportunities to interpret in the clinical setting
◯ Yes
◯ No

I estimate that between the class and testing, I practiced
◯ 5-10 hours total
◯ 10-20 hours total
◯ Other

How much? ________________________________

In addition the practicing interpretation, I also
☐ Worked on medical terms in my other language
☐ Worked on my general vocabulary in my other language
☐ Engaged in other skill building activities

what? ________________________________

The most valuable part of this training has been ________________________________

I would add the following to the training ________________________________

As a result of the training, I feel

confident about interpreting
◯ more
◯ less
◯ same

empathy for LEP patients
◯ more
◯ less
◯ same

empathy for English-only providers
◯ more
◯ less
◯ same

Inclined to advocate for patients
◯ more
◯ less
◯ same

Inclined to work for system change
- ◯ more
- ◯ less
- ◯ same

I would recommend this experience to fellow students
- ◯ Yes
- ◯ No

I would be willing to participate as a coach in future trainings
- ◯ Yes
- ◯ No
